# EGR2 is elevated and positively regulates inflammatory IFNγ production in lupus CD4^+^ T cells

**DOI:** 10.1186/s12865-020-00370-z

**Published:** 2020-07-09

**Authors:** Rujuan Dai, Bettina Heid, Xiguang Xu, Hehuang Xie, Christopher M. Reilly, S. Ansar Ahmed

**Affiliations:** 1 Department of Biomedical Sciences and Pathobiology, Virginia-Maryland College of Veterinary Medicine, Virginia Tech, Blacksburg, VA USA; 2 Department of Biological Sciences, Virginia Tech, Blacksburg, VA USA; 3 Fralin Life Sciences Institute at Virginia Tech, Blacksburg, VA USA; 4 Edward Via College of Osteopathic Medicine, Blacksburg, VA USA

**Keywords:** EGR2, Lupus, Inflammation, IFNγ, Th1

## Abstract

**Background:**

Recent studies have shown that early growth response 2 (EGR2) is highly induced in activated T cells and regulates T cell functions. In normal C57BL/6 (B6) mice, deletion of EGR2 in lymphocytes results in the development of lupus-like systemic autoimmune disease, which implies indirectly an autoimmune protective role of EGR2. Conversely, increased EGR2 gene expression is suggested to link with high risk of human lupus. In the present studies we sought to clarify the expression and inflammation regulatory role of EGR2 in murine lupus T cells directly.

**Results:**

We performed RT-qPCR analysis and found a significant increase of EGR2 mRNA expression in human lupus PBMCs and in CD4^+^ T cells from three different murine lupus models including MRL-*lpr*, B6-*lpr*, and B6.*sle123* mice at diseased stage when compared to age-matched control MRL or B6 mice. By performing intracellular flow cytometry analysis, we found that EGR2 protein expression was significantly increased in resting lupus (either MRL-*lpr* or B6.*sle123*) CD4^+^ T cells when compared to CD4^+^ T cells from their respective non-autoimmune controls. However, there was no difference of EGR2 protein expression in anti-CD3 and anti-CD28 stimulated control and lupus CD4^+^ T cells since there was a stronger induction of EGR2 in activated control CD4^+^ T cells. EGR2 expression was significantly increased in MRL-*lpr* mice at an age when lupus is manifested. To understand further the function of elevated EGR2 in lupus CD4^+^ T cells, we inhibited EGR2 with a specific siRNA in vitro in splenocytes from MRL-*lpr* and control MRL mice at 15 weeks-of-age. We found that EGR2 inhibition significantly reduced IFNγ production in PMA and ionomycin activated MRL-*lpr* lupus CD4^+^ T cells, but not control MRL CD4^+^ T cells. We also found that inhibition of EGR2 in vitro suppressed the Th1 differentiation in both MRL and MRL-*lpr* naïve CD4^+^ T cells.

**Conclusions:**

EGR2 is highly upregulated in human and murine lupus cells. Our in vitro data suggest a positive role of EGR2 in the regulation of Th1 differentiation and IFNγ production in lupus effector CD4^+^ T cells.

## Background

The early growth response (EGR) family members including EGR1, 2, 3, and 4 are immediate early response genes, which play important regulatory roles in the development and functions of various biological systems [1, 2]. Among them, EGR2 is induced by T cell receptor (TCR) engagement and is required for the induction of T cell anergy [3, 4]. EGR2 is highly induced in activated T cells to negatively regulate T cell activation to control overwhelming inflammation [5, 6]. The non-autoimmune wildtype C57BL/6 (B6) mice with EGR2 deficiency in both T and B cells (CD2-CreEGR2^−/−^) had late-onset (after 6 months old) lupus-like autoimmune disease, characterized by an accumulation of highly activated CD4^+^CD44^+^ T cells and infiltration of IFNγ- and IL-17- producing CD4^+^ T cells in multiple organs [5]. The CD2-CreEGR2^−/−^ mice were also more susceptible to experimental autoimmune encephalomyelitis (EAE) induction than wild type B6 mice as the result of enhanced Th17 differentiation and IL-17 production in these mice [7]. To determine the role of EGR2 specifically in T cells, Okamura et al. generated T cell specific EGR2 depletion B6 mice (CD4-CreEGR2^−/−^) and reported that EGR2 controls humoral immune responses and autoimmunity via regulating the function of TGFβ3-expressing CD4^+^CD25^−^LAG3^+^ Tregs [8]. The CD4-CreEGR2^−/−^ mice had a significant increase of T cell follicular cells (T_FH_) and germinal center B cells (GCB cells), and displayed a more robust B cell response with enhanced antibody production in response to the administration of 4-hydroxy-3-nitrophenylacetyl (NP)-OVA antigen [8].

The synergistic role for EGR2 and EGR3 in controlling inflammation has been reported. Conditional depletion of both EGR2 and EGR3 led to the development of early onset and much more severe autoimmune syndromes than depletion of EGR2 alone in B6 mice [9, 10]. It is notable that B6 mice with EGR2 and EGR3 deficiency only in T cells (CD4-CreEGR2^−/−^EGR3^−/−^) survived longer than the mice with EGR2 and EGR3 depletion in both T and B cells (CD2-CreEGR2^−/−^EGR3^−/−^), suggesting an important regulatory role of EGR2 and EGR3 in B cells [9, 10]. Together, the above studies imply a protective role of EGR2 in preventing the development of lupus-like systemic autoimmune conditions in normal B6 mice [5, 8–11].

The studies with CD2-CreEGR2^−/−^ mice demonstrated that EGR2 negatively regulated IFNγ and IL-17 production in activated CD4^+^ T cells, suppressed Th17 differentiation, but has no obvious effect on Th1 differentiation [5, 7]. In contrast, Du et al. generated CD4-CreEGR2^−/−^ mice and reported that EGR2 positively regulated naïve CD4^+^ T cells differentiation into Th17 and Th1 cells and also effector T cell responses [12]. The Th1/Th17 cells differentiated from EGR2 deficient naïve CD4^+^ T cells had a lower level of lineage cytokine expression than wild type Th1/Th17 cells [12]. Du et al. further demonstrated that EGR2 was required for effective normal T cell response to influenza infection in vivo. Compared to wild type T cells, EGR2 deficient T cells produced a lower level of inflammatory cytokines (such as IFNγ, IL-2, and TNFα). As a result, the CD4-CreEGR2^−/−^ mice had delayed viral clearance and severe lung pathology than wild type B6 mice [12]. On the other hand, Ramon et al. reported that EGR2 was not required for in vivo CD4^+^ T cell response to pathogenic infection with Toxoplasma gondii and choriomeningitis virus [13].

The studies with EGR2 in normal B6 mice indirectly imply an important role of EGR2 in the regulation of autoimmune conditions. However, thus far only limited studies have examined EGR2 expression and function directly in autoimmune conditions. One study reported that there was a lower EGR2 expression in anti-CD3 and anti-CD28 activated CD4^+^ T cells from human multiple sclerosis (MS) patients when compared to healthy controls, correlating with higher IL-17 production in these cells. However, the expression of EGR2 in resting CD4^+^ T cells was not different between MS patients and healthy controls [7]. Nevertheless, in other autoimmune conditions, especially rheumatic diseases, EGR2 may have a different role. Candidate gene association analysis revealed that a regulatory polymorphism in the EGR2 gene was associated with susceptibility to both rheumatoid arthritis (RA) and lupus, and that increased EGR2 expression may contribute to lupus pathogenesis [14]. Moreover, elevated EGR2 has been noted in the murine scleroderma and also in skin and lung biopsy specimens from patients with systemic sclerosis, an autoimmune disease that has overlapping symptoms with lupus [15].

Taken together, these studies suggest that the expression and function of EGR2 is context-dependent, and it likely varies at a different developmental stage and/or different pathological conditions. Therefore, to clarify the role of EGR2 in lupus, it is essential to investigate further the expression and function of EGR2 in the lupus setting. However, there is so far no comprehensive investigation of EGR2 expression and function directly in human lupus and/or in murine models of lupus. Accordingly, in this study, we analyzed the expression of EGR2 in human lupus patients and in three different murine lupus models and detailed further the role EGR2 plays in the regulation of CD4^+^ T cell response and Th1 differentiation in lupus-prone mice.

## Results

### EGR2 mRNA expression is highly upregulated in both human and murine lupus cells

**Fig. 1:**
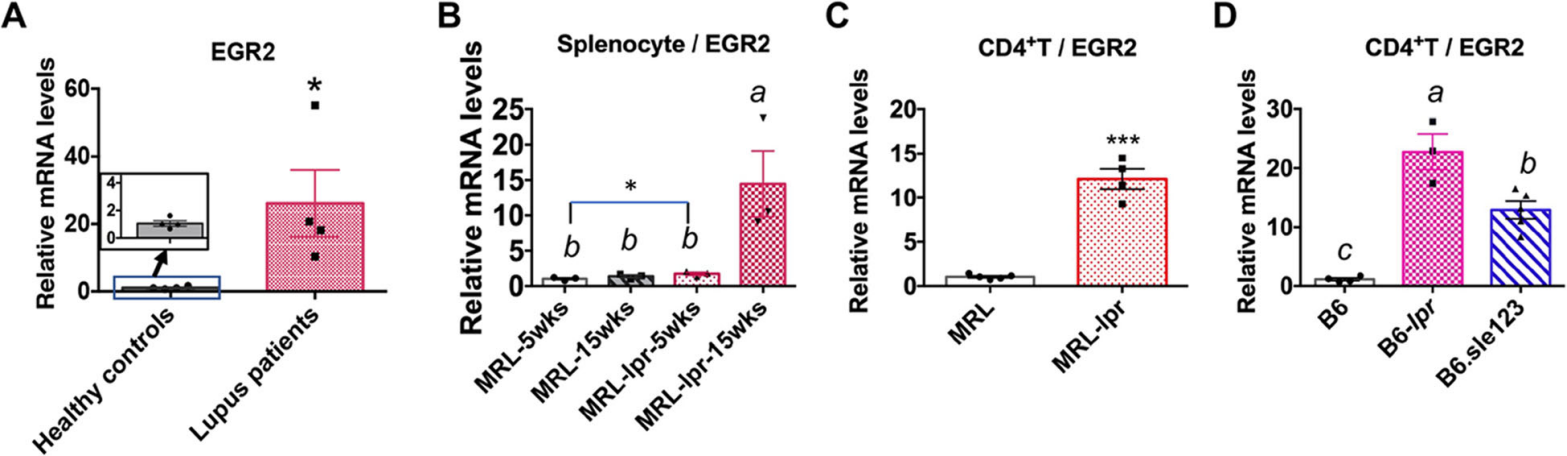
Increased EGR2 mRNA expression in human and murine lupus cells. (**a**) RT-qPCR analysis of EGR2 mRNA expression in human lupus and healthy control PBMCs samples. The graph shows means ± SEM (*n* = 4 each). (**b**) RT-qPCR analysis of EGR2 mRNA expression in whole splenocytes of 5 weeks old (representing pre-disease stage), 15 weeks old (representing disease stage) MRL-*lpr* and age-matched control MRL mice. The graph shows means ± SEM (*n* = 3 each). (**c**) EGR2 mRNA expression in purified splenic CD4^+^ T cells of 14–15-week-old MRL-*lpr* and control MRL mice. The graph shows means ± SEM (*n* ≥ 4). (**d**) EGR2 mRNA expression in purified splenic CD4^+^ T cells of disease B6-*lpr* (18-week-old) and B6.*sle123* (27–32-week-old) mice, and control B6 mice (27–32-week-old). The graph shows means ± SEM (*n* ≥ 3). Unpaired student t-tests were performed (lupus patients vs heathy controls, MRL vs MRL-*lpr*). *, *p* < 0.05, and ***, *p* < 0.001. One-way ANOVA with Tukey- Kramer all pair’s comparisons were performed for statistical analysis of multiple groups (**b** and **d**). The groups that were not connected with the same letter were significantly different in their means

Increased EGR2 expression is suggested to link with lupus susceptibility in humans [14]. We therefore performed RT-qPCR analysis to compare EGR2 expression in peripheral blood mononuclear cells (PBMCs) from human lupus patients and healthy controls (Fig. 1a). We found that EGR2 mRNA expression was significantly higher in human lupus PBMCs than healthy controls (Fig. 1a). We then analyzed EGR2 mRNA expression in splenocytes of MRL/MpJ-*Fas*^*lpr*^/J (MRL-*lpr*) mice at pre-diseased (5 weeks-of age) and diseased (15 weeks-of-age). As controls, we used aged matched MRL/MpJ (MRL) mice. Compared to either age-matched MRL control or pre-diseased MRL-*lpr* lupus mice, EGR2 expression was significantly increased in MRL-*lpr* mice at 15 weeks-of-age (Fig. 1b). There was also a slight but significant increase of EGR2 mRNA in splenocytes from MRL-*lpr* mice at 5 weeks-of-age when compared to age matched MRL controls (*p* = 0.04, MRL vs MRL-*lpr*, student *t* test). We next investigated whether EGR2 mRNA expression was upregulated in purified splenic CD4^+^ T cells from MRL-*lpr* mice as well as the other two different murine lupus stains B6.MRL-*Fas*^*lpr*^/J (B6-*lpr*) and B6.NZMSle1/Sle2/Sle3 (B6.*sle123*) mice at diseased stage. We found that EGR2 mRNA expression levels were significantly upregulated in purified splenic CD4^+^ T cells from diseased MRL-*lpr* (14–15 weeks-of-age, Fig. 1c), B6-*lpr* (18 weeks-of-age, Fig. 1d) and B6.*sle123* (27–32 weeks of age, Fig. 1d) lupus mice when compared to their respective controls (MRL and B6 mice). The development and progression of lupus in MRL-*lpr*, B6-*lpr*, and B6.*sle123* mice as they age has been previously reported [16, 17]. Together, our data revealed a common upregulation of EGR2 mRNA expression in human lupus and in different murine lupus models. To further investigate the role of EGR2 in lupus, we assessed the EGR2 expression in different splenic lymphocyte subsets in the MRL-*lpr* and B6.*sle123* models as these two models have different genetic contributions in the disease pathogenesis.

### Both the percentage of EGR2 expressing cells and EGR2 protein expression intensity are highly upregulated in splenic CD4^+^ T cells of MRL-*lpr* and B6.*sle123* lupus mice

**Fig. 2:**
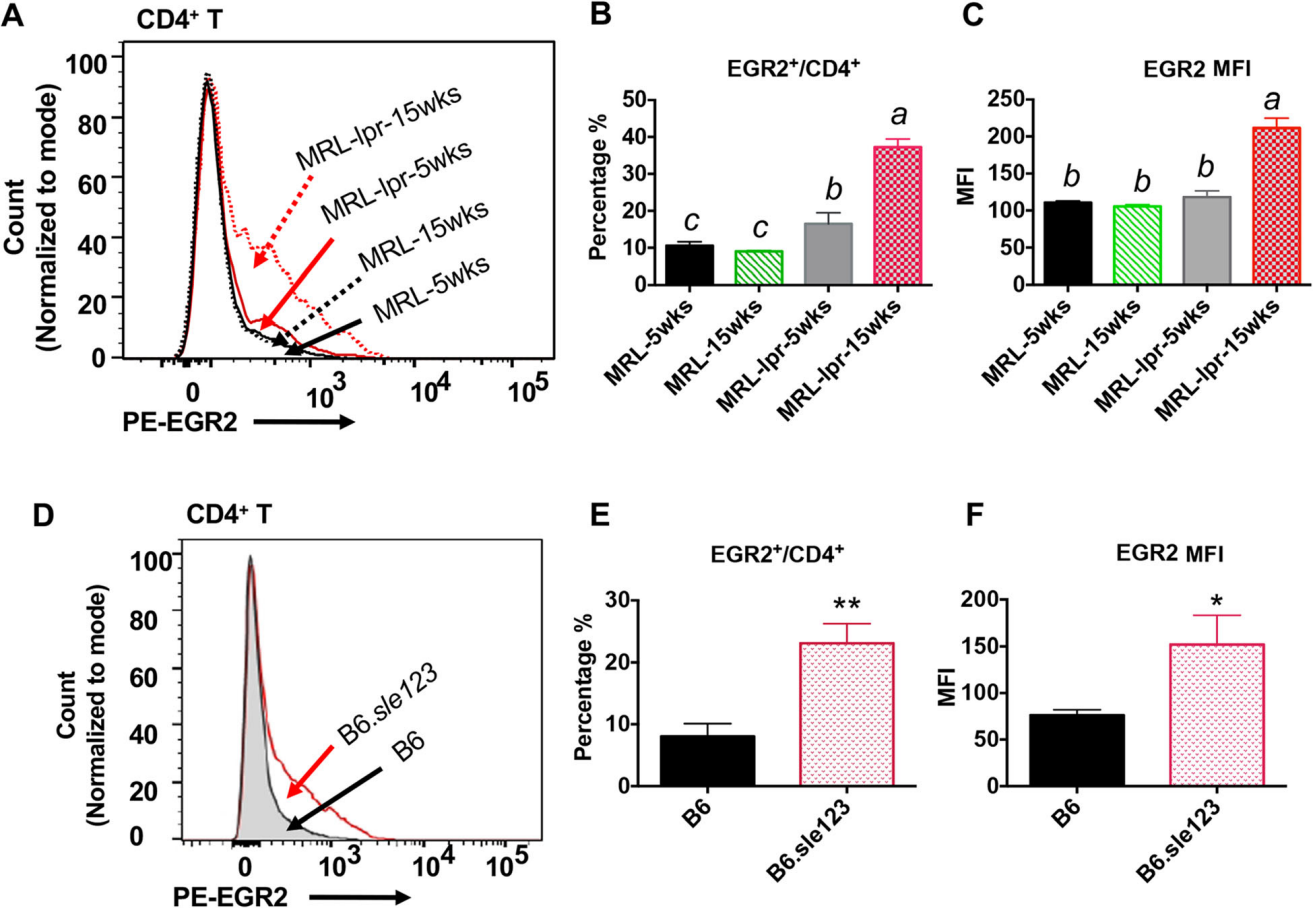
Increased percentage of EGR2 expressing cells and EGR2 expression intensity in splenic CD4^+^ T cells of MRL-*lpr* and B6.*sle12*3 mice. The freshly-isolated splenocytes were stained with cell surface marker CD4, and then subjected to intracellular flow stain of EGR2. (**a-c**) Intracellular flow cytometry analysis of EGR2 expression in gated splenic CD4^+^ T cells of MRL and MRL-*lpr* at 5 weeks-of age and15 weeks-of age. The representative histogram plot shows EGR2 protein expression in gated CD4^+^ T cells of MRL-*lpr* and control MRL mice at different age (**a**). The summary graphs show the percentage of EGR2 expressing cells in CD4^+^ T cells (EGR2^+^/CD4^+^, **b**) and EGR2 expression intensity (MFI, **c**) in MRL-*lpr* and MRL CD4^+^ T cells. Graphs show means ± SEM (*n* ≥ 4). (**d-f**) Intracellular flow cytometry analysis of EGR2 protein expression in gated splenic CD4^+^ T cells of B6 and B6.*sle123* lupus mice. The representative histogram plot shows EGR2 protein expression in gated splenic CD4^+^ T cells of B6.*sle123* (26–27-week- old) and age-matched control B6 mice. The summary graphs show the percentage of EGR2-expressing cells (**e**) and EGR2 MFI (**f**) in B6.*sle123* and control B6 CD4^+^ T cells. Graphs show means ± SEM (*n* = 3 each). One-way ANOVA with Tukey- Kramer all pair’s comparisons were performed for statistical analysis of multiple groups (**b** and **c**). The groups that were not connected with the same letter were significantly different in their means. Unpaired student *t*-tests (B6 vs B6.*sle123*); *, *p* < 0.05, and **, *p* < 0.01

**Fig. 3:**
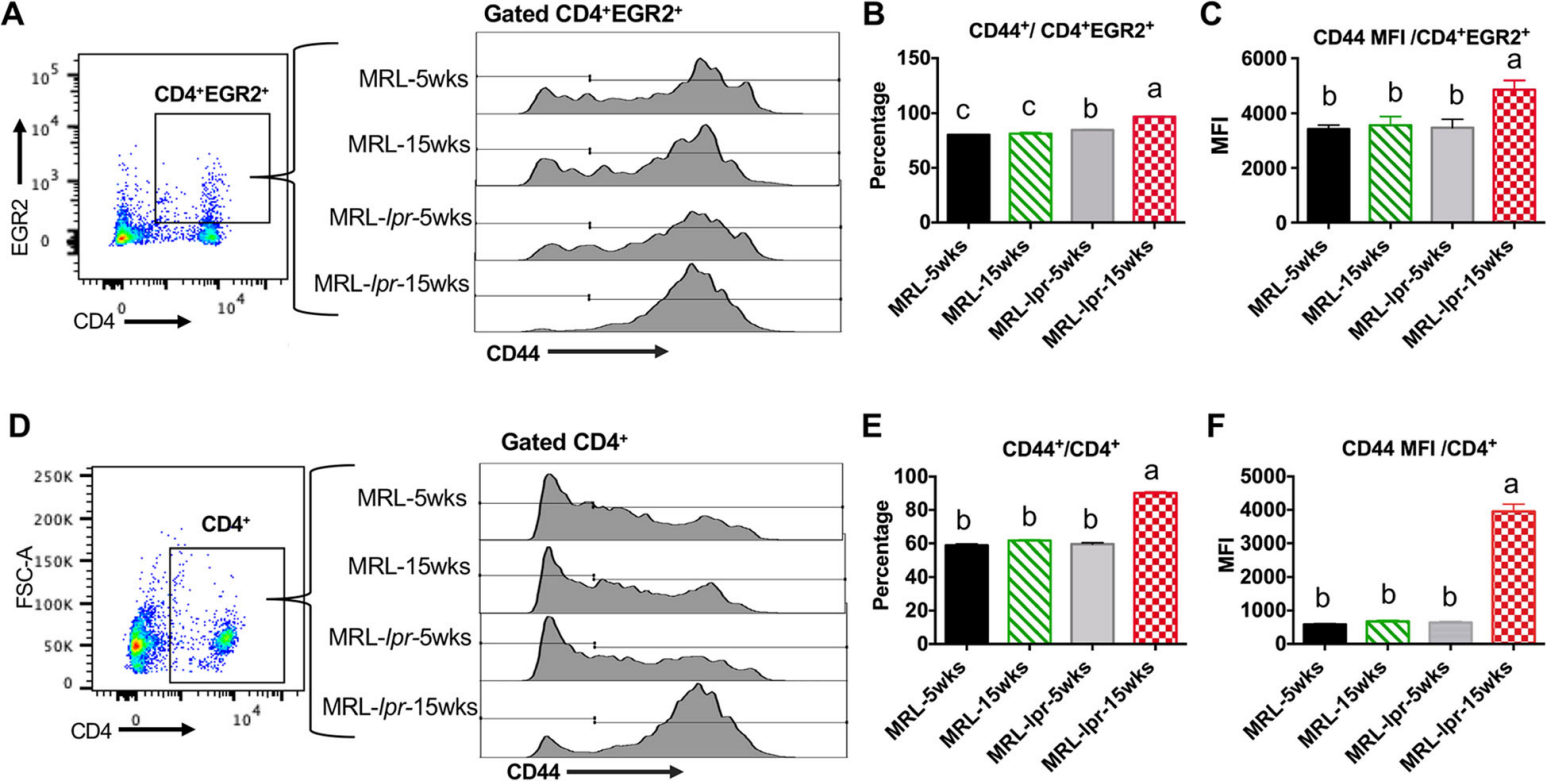
The increase of EGR2 expression in MRL-*lpr* lupus CD4^+^ T cells accompanies with increased T cell activation following disease development. The freshly-prepared splenocytes from 5-week-old and 15-week-old MRL an MRL-*lpr* mice were stained with cell surface marker CD4 and CD44, and then subjected to intracellular flow stain of EGR2. (**a**) The stained splenocytes were gated at CD4^+^EGR2^+^ cells to analyze the expression of CD44 in CD4^+^EGR2^+^ cells. The representative histogram plots show CD44 expression in gated CD4^+^EGR2^+^ cells of MRL-*lpr* and control MRL mice at different age. (**b** & **c**) The summary graphs show the percentage of CD44 expressing cells (CD44^+^/ CD4^+^EGR2^+^, b) and CD44 expression intensity (MFI, c) in gated CD4^+^EGR2^+^ cells. Graphs show means ± SEM (*n* ≥ 4). (**d**) The stained splenocytes were gated at CD4^+^ cells to analyze the expression of CD44 in CD4^+^ T cells. The representative histogram plots show CD44 expression in gated CD4^+^ cells of MRL-*lpr* and control MRL mice at different age. (**e** & **f**) The summary graphs show the percentage of CD44 expressing cells (CD44^+^/ CD4^+^, e) and CD44 expression intensity (**f**) in gated CD4^+^ cells. Graphs show means ± SEM (*n* ≥ 4). One-way ANOVA with Tukey- Kramer all pair’s comparisons were performed for statistical analysis. The means of groups that were not connected with the same letter were significantly different

We performed an intracellular flow cytometry assay to quantify EGR2 expressing cells and EGR2 protein expression intensity (determined by Median Fluorescence Intensity, MFI) in gated splenic CD4^+^ T cells of MRL-*lpr* and B6.*sle123* lupus mice and non-autoimmune controls (MRL and B6). Consistent with increased EGR2 mRNA expression in MRL-*lpr* CD4^+^ T cells (Fig. 1c), EGR2 protein expression (both EGR2^+^/CD4^+^ percentage and MFI) was significantly higher in CD4^+^ T cell from diseased MRL-*lpr* mice (15 weeks-of-age) when compared to either age matched control MRL mice or pre-diseased MRL-*lpr* mice (5 weeks-of-age) (Fig. 2a-c*).* The EGR2^+^/CD4^+^ percentage was increased in 5-week old MRL-*lpr* mice when compared to 5-week-old MRL controls (Fig. 2b), although the expression intensity was not different (Fig. 2c). Similar to that in MRL-*lpr* lupus mice, both the percentage of EGR2 expressing CD4^+^ T cells and EGR2 expression intensity were increased in B6. *sle123* lupus mice at moderate disease stage (26–27 weeks-of-age) when compared to age matched control B6 mice (Fig. 2d-f). Consistent with the previous report that EGR2 is mainly expressed in activated CD4^+^ T (CD44^+^CD4^+^) cells, we found that majority (over 80% of EGR2^+^CD4^+^ T in MRL and MRL-*lpr* were CD44^+^ T cells (Fig. 3a-c). The increase of EGR2 expressing CD4^+^ T cells in MRL-*lpr* mice was associated with a increased percentage of activated CD4^+^ T cells (CD44^+^/CD4^+^) (Fig. 3d-f). There was a significantly higher percentage of CD44 expressing CD4^+^ T cells and CD44 expression intensity in gated CD4^+^ T cells of diseased MRL-*lpr* mice (15 weeks-of-age) (Fig. 3e and f). Together, our data demonstrated a significant increase of EGR2 expression in CD4^+^ T cells of murine lupus cells when compared to their respective controls.

### T cell activation induces EGR2 protein expression in different splenic cell subsets of lupus mice (MRL-*lpr* and B6.*sle123)* and controls (MRL and B6)

**Fig. 4:**
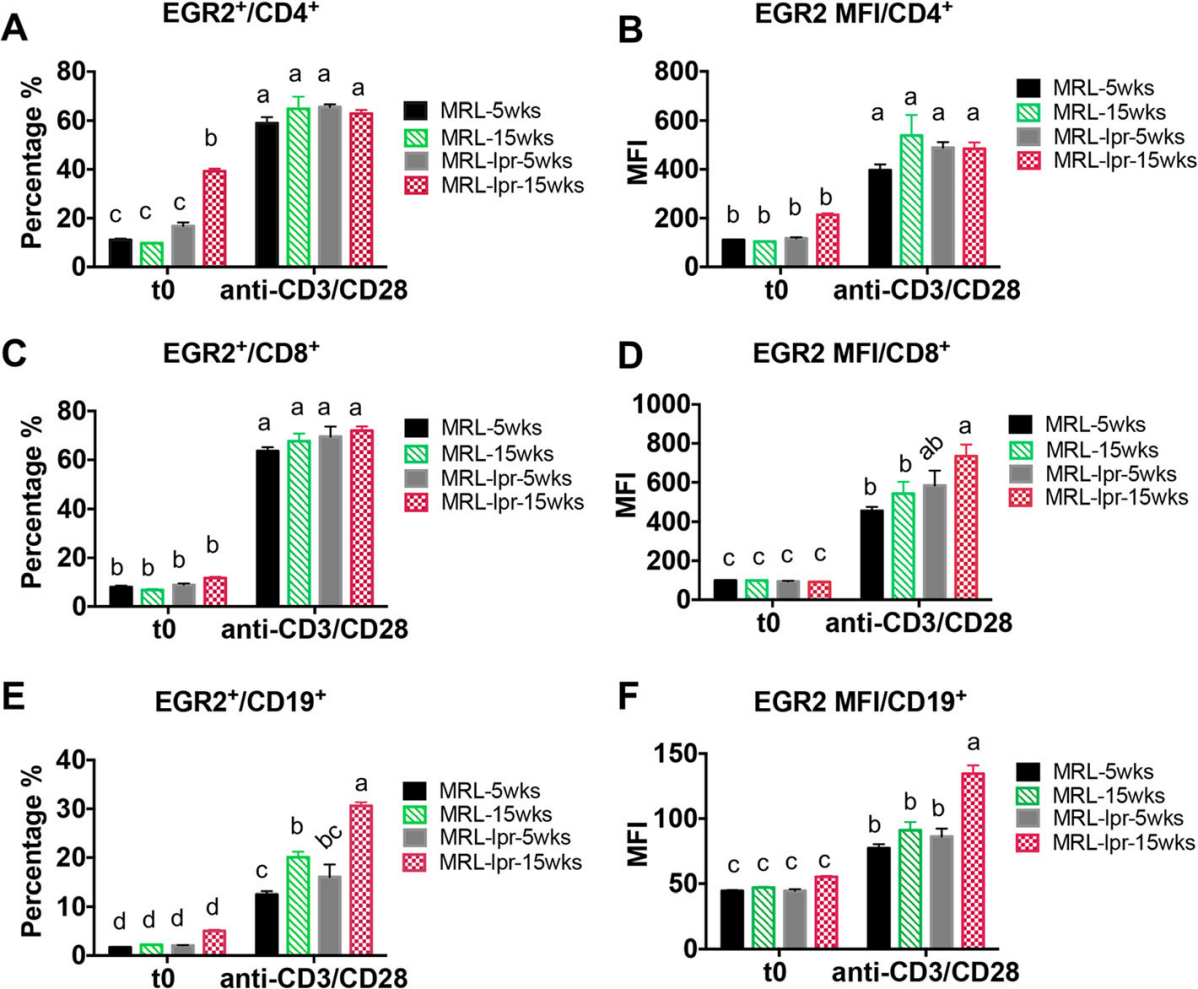
T cell activation induces EGR2 expression significantly in T and B lymphocytes of both MRL and MRL-*lpr* mice. The freshly-prepared (t0, unstimulated cells), anti-CD3 and anti-CD28 stimulated (24 h of stimulation) splenocytes from 5-week-old and 15-week-old, MRL and MRL-*lpr* mice were stained with different cell surface marker (CD4, CD8, CD19, B220, CD3) and then subjected to intracellular flow stain of EGR2. The cells were gated on CD4^+^ T, CD8^+^ T, and CD19^+^B cells respectively to analyze the expression of EGR2. The graphs show the percentage EGR2 expressing cells and EGR2 expression intensity (MFI) in CD4^+^ T (**a **& **b**), CD8^+^ T (**c **& **d**), CD19^+^ B (**e **& **f**) cells. Graphs show means ± SEM (*n* ≥ 4). One-way ANOVA with Tukey- Kramer all pair’s comparisons were performed for statistical analysis. The means of groups that were not connected with same letter were significantly different

Next, we evaluated the expression of EGR2 in different splenic lymphocyte subsets (CD4^+^ T, CD8^+^ T, CD19^+^ B cells) at resting and activated state in MRL and MRL-*lpr* mice at pre- and active disease stage. At resting state (t0, representing freshly-isolated and unstimulated cells), we only observed a significant increase of the percentage of EGR2 expressing cells in CD4^+^ T cells (EGR2^+^/CD4^+^), but not in either the CD8^+^ T cells (EGR2^+^/CD8^+^) nor the CD19^+^ B cells (EGR2^+^/CD19^+^) from 15-week-old MRL-*lpr* mice (Fig. 4a, c and e). Following T cell activation with anti-CD3 and anti-CD28 stimulation of splenocytes for 24 h, EGR2 expression (both percentage and MFI) was significantly increased in T and B cells when compared to that at resting state (Fig. 4a-f). Interestingly, at the activation state, there was no difference in EGR2 expression in CD4^+^ T cells between MRL and MRL-*lpr* mice at different ages (Fig. 4a & b). For CD8^+^ T cells, we observed only an increase of EGR2 expression intensity in activated CD8^+^ T cells from 15-week-old MRL-*lpr* mice when compared to MRL mice (Fig. 4d). Compared to CD4^+^ and CD8^+^ T cells, CD19^+^ B cells had much lower EGR2 expression (both percentage and MFI). While there was no difference in the EGR2 expression at resting B cells, anti-CD3 and anti-CD28 stimulation induced higher EGR2 expression (both EGR2^+^/CD19^+^ percentage and MFI) in B cells of diseased MRL-*lpr* (15 weeks-of-age) when compared to MRL (either 5 weeks-of age or 15 weeks-of-age) or pre-diseased MRL-*lpr* (5 weeks-of age) mice (Fig. 4e & f).

**Fig. 5:**
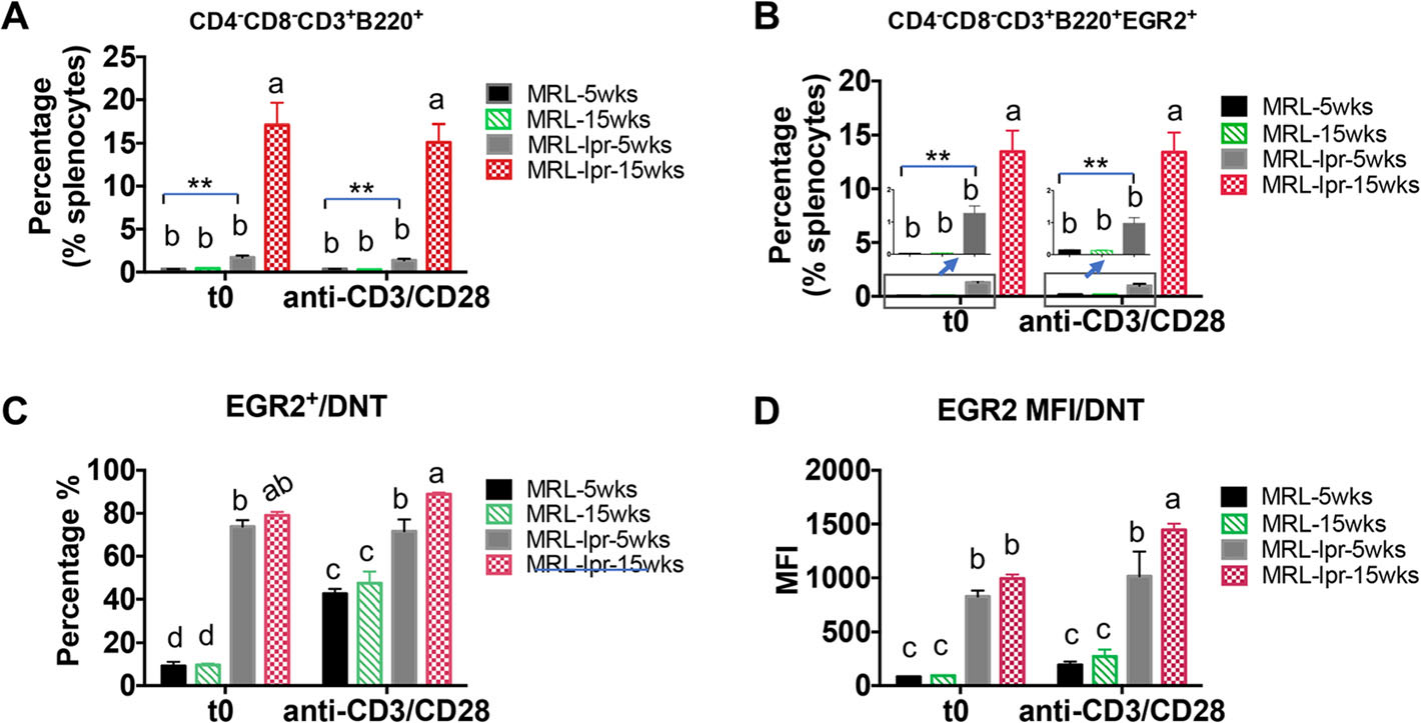
T cell activation has limit effect on the induction of EGR2 in DNT cells of MRL-*lpr* mice. The splenocytes were prepared and stained as described in Fig. 4 legend. The cells were firstly gated on CD4^−^CD8^−^ cells, and then gated on CD3^+^B220^+^ cells to identify the percentage of DNT (CD4^−^CD8^−^CD3^+^B220^+^) cells (**a**) and EGR2 expressing DNT cells in whole splenocytes (**b**). The graphs show the summary of the percentage of EGR2 expression (**c**) and EGR2 expression intensity (MFI) (**d**) in gated DNT cells from MRL and MRL-*lpr* splenocytes at resting and activation state. Graphs show means ± SEM (*n* ≥ 4). One-way ANOVA with Tukey- Kramer all pair’s comparisons were performed for statistical analysis. The groups that were not connected with the same letter were significantly different in their means

A unique feature of *lpr* lupus mice is the development and accumulation of CD4^−^CD8^−^CD3^+^B220^+^ (double negative T or DNT) cells, which is attributed to the *fas* gene mutation [18]. Compared to MRL mice, the percentage of DNT cells in splenocytes of MRL-*lpr* mice was slightly increased even before the onset of lupus (5 weeks-of age), and was further increased dramatically in diseased MRL-*lpr* mice (15 weeks-of-age) (Fig. 5a). The percentage of EGR2 expressing DNT in whole splenocytes (EGR2^+^CD4^−^CD8^−^CD3^+^B220^+^/splenocytes) was also increased at a similar pattern as percentage of DNT cells in MRL-*lpr* mice (Fig. 5b). It is noteworthy that about 80% DNT cells in MRL-*lpr* mice (either 5 weeks-of-age or 15 weeks-of-age) were EGR2^+^ cells at the resting state (Fig. 5c). While anti-CD3 and anti-CD28 stimulation significantly increased EGR2^+^ cells in DNT cells of MRL mice, the stimulation did not induce a further increase of EGR2^+^ cells in DNT cells (EGR2^+^/DNT) of MRL-*lpr* mice (Fig. 5c). We only observed an increase of EGR2 expression intensity in DNT cells of activated splenocytes from 15-week-old MRL-*lpr* mice when compared to resting cells (Fig. 5d). These data indicate a strong and saturated expression of EGR2 in DNT cells of MRL-*lpr* mice, even in unstimulated cells from pre-diseased mice.

**Fig. 6:**
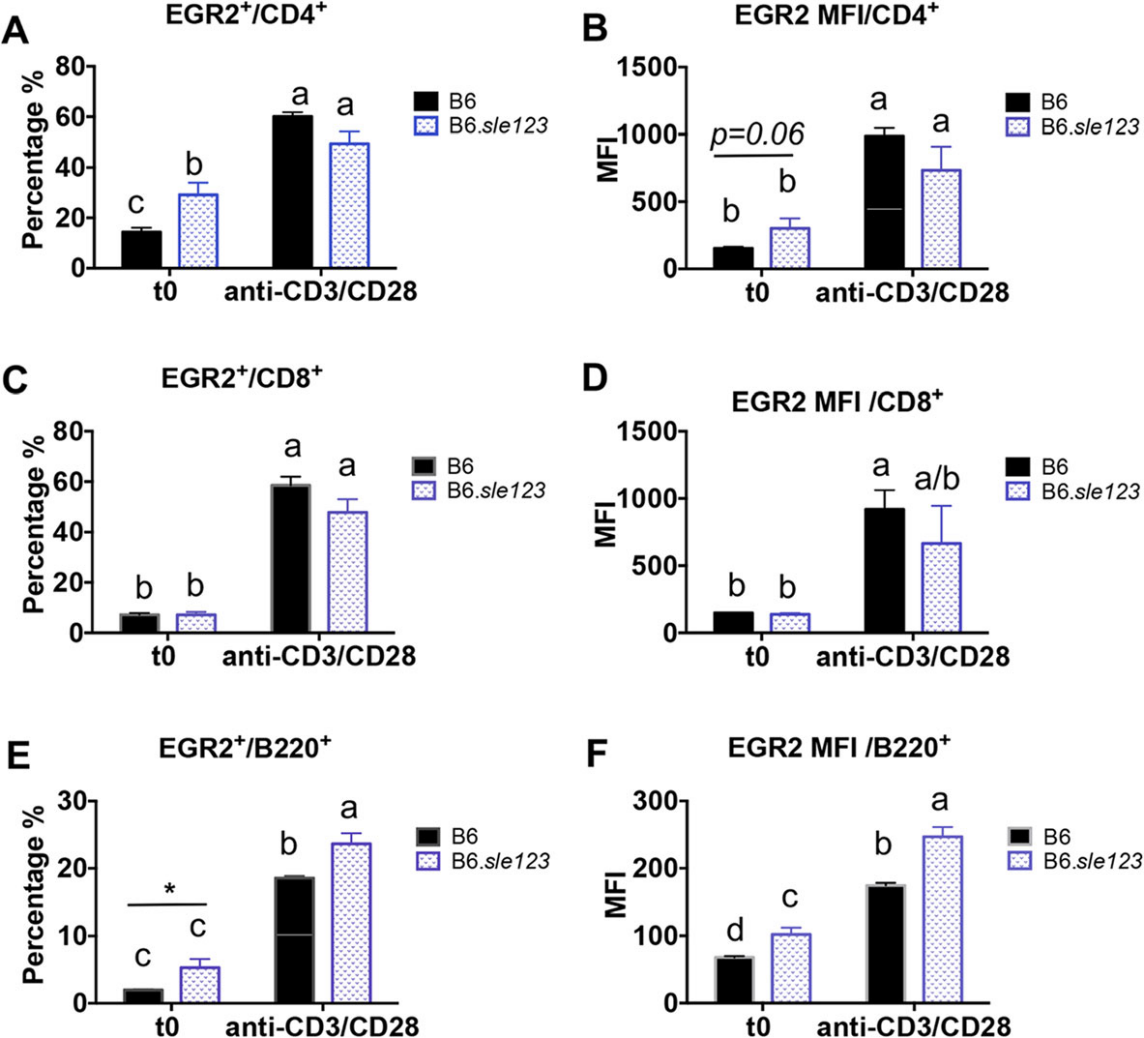
T cell activation induces EGR2 expression in different lymphocyte subsets of both B6 and B6.*sle123* mice. The freshly-prepared (t0), anti-CD3and anti-CD28 stimulated (24 h of stimulation) splenocytes from B6.*sle123* and control B6 mice (31–32-week-old) were stained with cell surface markers CD4, CD8, B220, and then subjected to intracellular flow stain of EGR2. The cells were gated on CD4^+^ T, CD8^+^ T, and B220^+^ B cells respectively to analyze the expression of EGR2. The graphs show the percentage of EGR2 expressing cells and EGR2 expression intensity (MFI) in CD4^+^ T (**a **& **b**), CD8^+^ T (**c **& **d**), B220^+^ B (**e **& **f**) cells. Graphs show means ± SEM (*n* ≥ 4). One-way ANOVA with Tukey- Kramer all pair’s comparisons were performed for statistical analysis. The groups that were not connected with the same letter were significantly different in their means

We also analyzed the expression of EGR2 in different splenic cell subsets of a different murine lupus strain, B6.*sle123* mice at resting state and following anti-CD3 and anti-CD28 stimulation. Similar to the findings in MRL-*lpr* lupus mice, at resting state, the percentage of EGR2 expressing cells was significantly increased in CD4^+^ T cells (EGR2^+^/CD4^+^), but not in CD8^+^ T cells from B6.*sle123* mice (31–32 weeks-of age) when compared to age-matched B6 controls (Fig. 6a, c, e). However, we observed an increase of EGR2 expression in B220^+^ B cells of B6.*sle123* mice at the resting state (Fig. 6e & f). Anti-CD3 and anti-CD28 stimulation highly induced EGR2 expression in splenic T and B cell subsets of both B6.*sle123* and control B6 mice (Fig. 6). Following the stimulation with anti-CD3 and anti-CD28, there was no difference of the percentage of EGR2 expressing cells in CD4^+^ and CD8^+^ T cells between B6. *sle123* and control B6 mice, but there was a higher EGR2 expression in splenic B cells of B6. *sle123* when compared to B6 controls (Fig. 6e, f). Together, our data suggested that EGR2 expresses at a low level in the resting T and B lymphocytes and that anti-CD3 and anti-CD28 stimulation significantly induces EGR2 expression in splenic T and B lymphocytes in both control and lupus mice.

### Inhibition of EGR2 in vitro reduces IFNγ production in splenic CD4^+^ T cells of MRL-*lpr* lupus mice

**Fig. 7:**
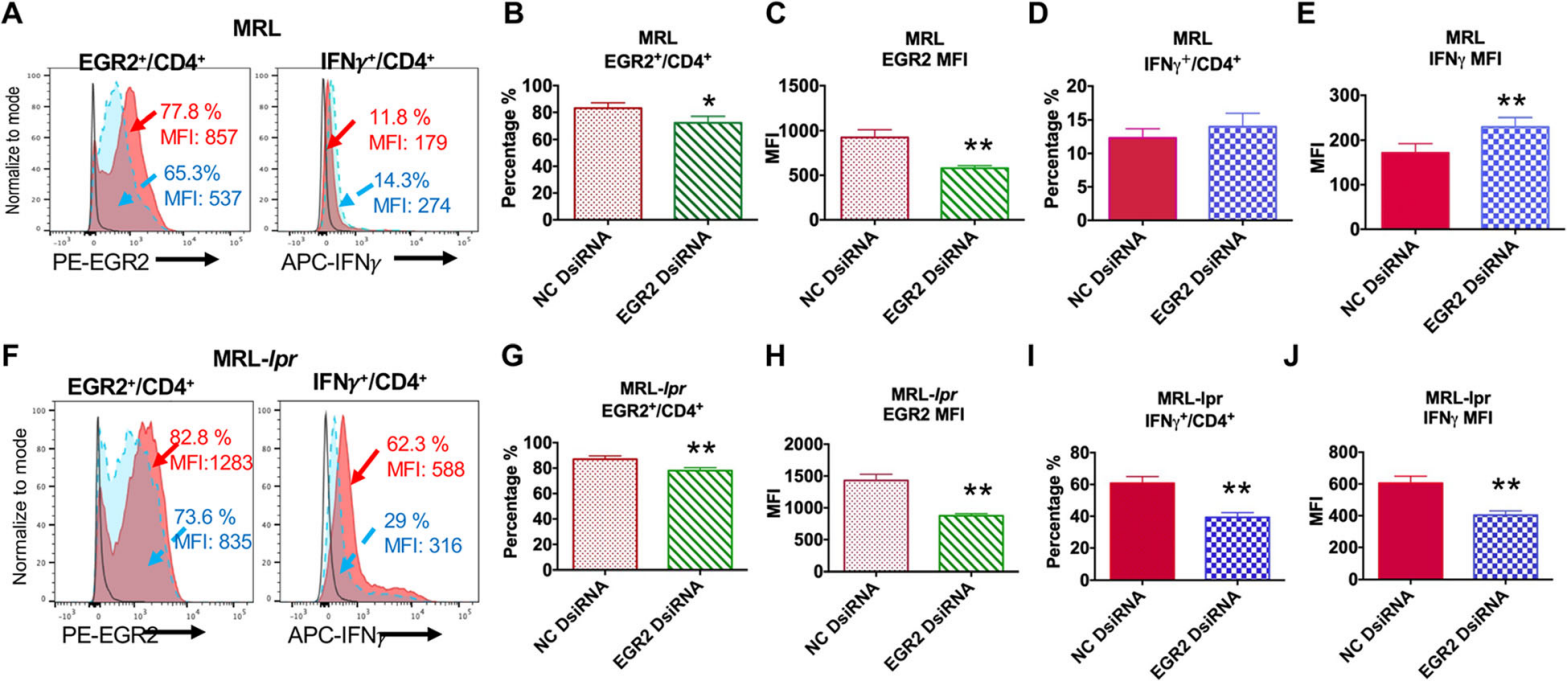
Inhibition of EGR2 significantly reduces IFNγ production in splenic CD4^+^ T cells of MRL-*lpr* lupus, but not control MRL mice. The splenocytes form 14–15-week- old MRL and MRL-*lpr* mice were transfected with negative control (NC, solid Red) and EGR2 specific (dotted blue) DsiRNA, respectively. Forty-eight hours after transfection, the cells were stimulated with PMA and ionomycin and Golgi protein transport inhibitor to analyze the expression of IFNγ and EGR2 in gated CD4^+^ T cells by intracellular flow cytometry. The solid black line represents the baseline EGR2 and IFNγ expression in unstimulated cells. (**a**) The representative histogram plots show the EGR2 and IFNγ expression in gated CD4^+^ T cells of NC and EGR2 DsiRNA transfected MRL splenocytes. (**b-e**) The summary graphs show the percentage of EGR2 expressing cells (**b**), EGR2 expression intensity (MFI, **c**), the percentage of IFNγ expressing cells (**d**), IFNγ expression intensity (**e**) in gated CD4^+^ T cells of NC and EGR2 DsiRNA treated MRL splenocytes. Graphs show means ± SEM (*n* = 6 each). (**f**) The representative histogram plots show the EGR2 and IFNγ expression in gated CD4^+^ T cells of DsiRNA transfected MRL-*lpr* splenocytes. (**g-j)** The summary graphs show the percentage of EGR2 expressing cells (**g**), EGR2 expression intensity (**h**), the percentage of IFNγ expression cells (**i**), IFNγ expression intensity (**j**) in gated CD4^+^ T cells of NC and EGR2 DsiRNA treated MRL-*lpr* splenocytes. Graphs show means ± SEM (*n* = 6 each). Paired student *t*-tests (NC vs EGR2 DsiRNA); *, *p* < 0.05, **, *p* < 0.01, and ***, *p* < 0.001

To understand the role of increased EGR2 in the context of lupus CD4^+^ T cells, we transfected splenocytes from MRL and MRL-*lpr* mice at 14–15 weeks-of age with specific EGR2 Dicer substrate siRNA (DsiRNA) to block EGR2 expression. The transfected cells were then stimulated with PMA and ionomycin to assess IFNγ production specifically in gated CD4^+^ T cells. PMA and ionomycin stimulation induced EGR2 and IFNγ expression in gated CD4^+^ T cells of both MRL and MRL-*lpr* mice when compared to their unstimulated controls (Fig. 7a, f).

In the stimulated splenocytes that received EGR2 DsiRNA treatment, EGR2 expression (both the percentage of EGR2 expressing cells (EGR2^+^/CD4^+^) and EGR2 MFI) in gated CD4^+^ T cells was significantly reduced when compared to negative control (NC) DsiRNA treated cells (Fig. 7b, c, g, h). Consistent with the published data demonstrating a negative regulatory role of EGR2 in B6 CD4^+^ T cells, inhibition of EGR2 increased IFNγ expression intensity in gated CD4^+^ T cells from control MRL mice, although it had no significant effect on the percentage of IFNγ-producing cells (Fig. 7d, e). Interestingly, there was a significant reduction of IFNγ (both the percentage of IFNγ^+^ expressing cells (IFNγ^+^/CD4^+^) and IFNγ MFI) in gated CD4^+^ T cells of EGR2 DsiRNA transfected MRL-*lpr* splenocytes when compared to NC DsiRNA transfected MRL-*lpr* splenocytes (Fig. 7i, j). Together, our data suggest that EGR2 positively regulates IFNγ production in MRL-*lpr* lupus CD4^+^ T cells, but not in control MRL CD4^+^ T cells.

### EGR2 regulates Th1 differentiation of naïve CD4^+^ T cells from both MRL and MRL-*lpr* mice

**Fig. 8:**
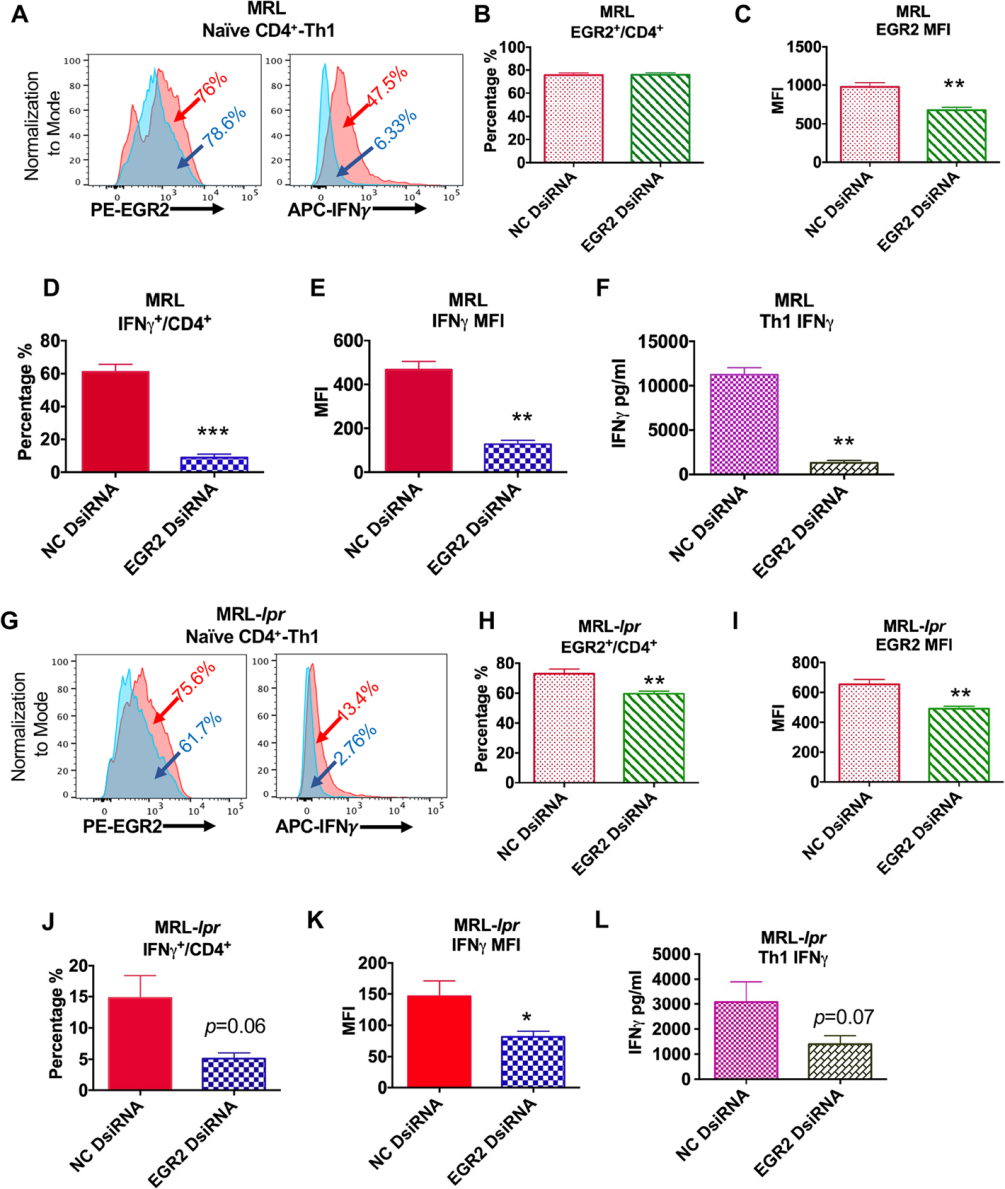
Inhibition of EGR2 suppresses Th1 differentiation of naïve CD4^+^ T cells from both MRL and MRL-*lpr* mice. Naïve CD4^+^ T cells from MRL and MRL-*lpr* mice at 6–8-week-old were transfected with NC (red) and EGR2 (blue) dsiRNA, respectively. Twenty-four hours after transfection, the cells were cultured at Th1 condition for 3 days. The Th1 cell culture supernatant were collected for ELISA; the cells were stimulated with PMA and ionomycin for 5 h for intracellular flow analysis. (**a & g**) The representative histogram plots show the EGR2 and IFNγ expression in the Th1 cells differentiated from NC and EGR2 DsiRNA treated MRL (**a**) and MRL-*lpr* (**g**) naïve CD4^+^ T cells. (**b-e and h-k**) The summary graphs show the percentage of EGR2 expressing cells (**b, h**), EGR2 expression intensity (**c & i**), the percentage of IFNγ expressing cells (**d, j**), IFNγ expression intensity (**e, k**) in Th1 cells differentiated from DsiRNA treated MRL and MRL-*lpr* naïve CD4^+^ T cells. (**f & l**) ELISA analysis of IFNγ in Th1 culture supernatant of DsiRNA-treated MRL (**f**) and MRL-*lpr* (**l**) naïve CD4^+^ T cell. Graphs show means ± SEM (*n* = 4 each). Paired student *t*-tests (NC vs EGR2 DsiRNA); *, *p* < 0.05, **, *p* < 0.01, and ***, *p* < 0.001

Consistent with the previous report showing that EGR2 is critical for Th1 differentiation [12], here, we found that inhibition of EGR2 in vitro with DsiRNA significantly suppressed Th1 polarization of naïve CD4^+^ T cell from both MRL and MRL-*lpr* mice at 6–8 weeks-of-age (Fig. 8a, g). EGR2DsiRNA treatment significantly reduced EGR2 expression intensity in Th1 cells differentiated from either MRL or MRL-*lpr* naïve CD4 T cells and also decreased EGR2^+^/CD4^+^ percentage from Th1 cells of MRL-*lpr* mice (Fig. 8b, c, h, j). Compared to the Th1 cells differentiated from NC DsiRNA transfected naïve CD4^+^ T, there was a significant reduction of both percentage of IFNγ-producing cell and expression intensity in the Th1 cells differentiated from EGR2 DsiRNA transfected naïve CD4^+^ T cells from either MRL or MRL-*lpr* mice (Fig. 8d, e, j, k). There was also reduced IFNγ levels in Th1 cell culture medium of EGR2 DsiRNA transfected cells when compared to NC DsiRNA controls (Fig. 8f, l). These data suggest a positive role of EGR2 in the regulation of naïve CD4^+^ T cells from both MRL and MRL-*lpr* mice differentiation into Th1 cells.

## Discussion

Emerging studies on EGR2 now indicate its role in the regulation of various immune cell development and functions. It is clear that EGR2 is highly induced in activated T cells to control T cell responses. However, the precise role of EGR2 in Th1 differentiation and regulating IFNγ production is unclear with conflicting data from different conditional EGR2 knock out B6 mouse models [7, 12, 13, 19]. In part, the conflicting findings on EGR2 regulation of Th1 differentiation and IFNγ production may be due to differences in studies which depleted EGR2 in different cell type (T and B cells for CD2-Cre vs T cells for CD4-Cre) CD2, and at different T cell development stage (double negative stage for CD2-Cre vs double positive stage for CD4-CRE) [5, 7, 12]. Additionally, it is likely that EGR2 function may be context-dependent (physiological vs pathological) [12, 13, 19]. In our studies, we showed EGR2 expression is upregulated in human and murine lupus (Figs. 1 and 2). The increase of EGR2 in lupus T cells might be a consequence of an inflammatory milieu with heightened T cell activation (CD44^+^CD4^+^) following lupus development. We further demonstrated that EGR2 plays a differential role in a lupus context since it positively regulated Th1 differentiation and IFNγ production in CD4^+^ T cells from MRL-*lpr* lupus mice (Figs. 7 and 8).

In our studies, we analyzed and compared the expression of EGR2 in resting and activated CD4^+^ T, CD8^+^ T, B cells of lupus and control mice (Figs. 4 and 6). At the resting state, we observed a significant increase of EGR2 in CD4^+^ T cells, but not in CD8^+^ T and CD19^+^ B cells of MRL-*lpr* mice (Fig. 4). The increase of EGR2 in CD4^+^ T cells may reflect the higher rate of activated CD4^+^ T cells (CD44^+^CD4^+^) in lupus mice (Fig. 3). It is interesting to note that the hierarchy of EGR2 expression intensity in different immune cell subsets was altered at the resting and activated state in diseased MRL-*lpr* (15 weeks-of age) and control MRL mice. For both MRL and MRL-*lpr* mice, EGR2 expression in B cells was the lowest at either resting state or activated state when compared to CD4^+^ T, CD8^+^ T and DNT cells (Supplemental Figure 1). For MRL mice, at the resting state, the EGR2 expression was comparable in CD4^+^ T, CD8^+^ T, and DNT cells (CD4^+^T ≈ CD8^+^ T ≈ DNT > CD19^+^B; Supplemental Fig. 1a,b). At the activation state, the hierarchy of EGR2 expression in MRL mice changed to CD4^+^T ≈ CD8^+^T > DNT > CD19^+^B (Supplemental Figure 1A &C). For MRL-*lpr* mice, at the resting state, EGR2 expression order was DNT > CD4^+^ T > CD8^+^ T > CD19^+^B (Supplemental Fig. 1d,e ). At the activation state, the hierarchy of EGR2 expression in MRL-*lpr* mice changed to DNT > CD8^+^ T > CD4^+^ T > CD19^+^B (Supplemental Fig.1d,f ). Similar results were observed for B6 and B6.*sle*123 mice (Supplemental Fig. 2). The lowest EGR2 expression was observed in B cells from both B6 and B6.*sle123* mice. While there was lower EGR2 in resting CD8^+^ T cells than that in resting CD4^+^ T cells of B6.*sle123* (Supplemental Figure 2C), there was comparable EGR2 expression in activated CD8^+^ T cells and CD4^+^ T cells of B6.sle123 (Supplemental Fig. 2d). Together, these data indicated that CD8^+^ T cells have a stronger response to anti-CD3 and anti-CD28 stimulation with regard to EGR2 induction in both MRL-*lpr* and B6.*sle123* lupus mice. Both CD4^+^ and CD8^+^ T cells contributed to the overproduction of inflammatory cytokine IFNγ in human lupus patients [20]. Compared to CD4^+^ T cells, there is much less investigation on the role CD8^+^ T cells in autoimmune diseases. Interestingly, we found increased EGR2 expression in B cells following T cell activation in splenocytes from both lupus (MRL-*lpr* and B6.*sle123)* and control (MRL and B6) mice, and that at the activation state, EGR2 expression was significantly higher in lupus B cells when compared to their respective controls (Figs. 4 and 6 e, f). It is possible that anti-CD3 and anti-CD28 activated T cells contributed to the increased EGR2 expression in B cells from lupus mice MRL-*lpr* mice either through the release of cytokines or co-stimulatory signals. DNT cells abnormally accumulate in *lpr* lupus mice as the result of *Fas* gene mutation and contribute to the lymphadenopathy and splenomegaly in MRL-*lpr* mice [18]. EGR2 expression levels in DNT cells of MRL-*lpr* mice were the highest when compared to CD4^+^ T, CD8^+^ T and B cells (Supplemental Fig. 1d-f). Almost 80% of DNT cells at resting state were EGR2^+^ cells in MRL-*lpr* mice (Fig. 5c). Anti-CD3 and antiCD28 stimulation increased EGR2 expression (both the percentage and expression intensity) in DNT cells of MRL mice, but only intensity in DNT cells of diseased MRL-*lpr* mice (Fig. 5c, d). This suggests that the expression level of EGR2 in MRL-*lpr* DNT cells was almost saturated at the resting state. An earlier study has reported that EGR2 was upregulated in the DNT cells of MRL-*lpr* (Fas^*lpr*^ mutation) and C3H-gld/gld (Fasl^gld^ mutation) lupus mice and bind to Fas ligand regulatory element (FLRE) to upregulate expression of Fas ligand (FasL) [21]. However, depletion of EGR2 in vivo did not affect the expression of FasL in B6 mice [5]. The function of highly elevated EGR2 in DNT cells in *lpr* mice remains to be elucidated.

Heightened Th1 cytokine IFNγ expression has been identified in both human and murine lupus and implicated in lupus pathogenesis [20, 22, 23]. While the administration of IFNγ accelerated lupus disease and increased mortality in NZB/W_F1_ mice, blocking IFNγ signaling with a specific antibody to IFNγ delayed the disease development and increased survival rate in NZB/W_F1_ mice [24]. Further support for the decisive role of IFNγ in murine lupus is demonstrated by the findings that depletion of IFNγ or IFNγ receptor in MRL-*lpr* and NZBW_F1_ mice reduced autoantibody production, improved histopathologic scores of kidneys, and promoted the survival of these lupus mice [25–27]. By utilizing MRL-*lpr* lupus model, we therefore investigated the role of EGR2 in the regulation of Th1 differentiation and Th1 cytokine IFNγ in the context of lupus. Consistent with the previous report showing that EGR2 positively regulate Th1 differentiation and IFNγ production in effector T cells [12], our in vitro study demonstrated a positive role of EGR2 in the regulation of IFNγ production in activated lupus CD4^+^ T cells and Th1 differentiation of both MRL-*lpr* lupus and control MRL naïve CD4^+^ T cells (Figs. 7 and 8). However, this finding is in contrast to the reports from Dr. Wang’s group demonstrating negative regulatory role EGR2 in controlling CD4^+^ T cell activation [5, 9]. It has been reported that in response to PMA and ionomycin stimulation, EGR2 deficient CD4^+^ T cells produced higher level of IFNγ than wild type CD4^+^ T cells from B6 mice [5]. It is noteworthy that our study was performed in vitro with lupus CD4^+^ T cells. Whether and how the upregulated EGR2 contributes to CD4^+^ T cell-mediated inflammation of autoimmunity in lupus needs to be further investigated, particularly in vivo in lupus mice with EGR2 deficiency. In addition, EGR2 has also been reported to critically regulate Th17 cell differentiation and IL-17 expression, either negatively or positively in different studies [7, 12]. Moreover, other than its suppressive role on T cell-mediated inflammation, EGR2 has also been shown to control autoimmunity by regulating the function of FoxP3 independent CD4^+^CD25^−^LAG3^+^ Treg cells [11, 28]. CD4^+^CD25^−^LAG3^+^ Tregs produce high levels of suppressive cytokine IL-10 and TGFβ-3. Further studies revealed that CD4^+^CD25^−^LAG3^+^ Tregs control B cell-mediated humoral immunity through EGR2- and Fas-dependent expression of TGFβ-3. While EGR2^+^ Fas^+^ LAG3^+^ Tregs were able to suppress lupus development in MRL-*lpr* mice, neither EGR2 or Fas deficient LAG3^+^ Tregs had autoimmune suppressive role [8]. To gain a better understanding of the function of elevated EGR2 in lupus, we think it is important and necessary in future studies to investigate EGR2 regulation in different immune cell subsets (such as CD4^+^ T cells, CD8^+^ T cells, B cells, Tregs, et al) directly in a lupus context by developing specific conditional EGR2 depletion murine lupus models.

## Conclusions

Overall, our studies demonstrated that EGR2 is significantly upregulated in human and murine lupus. Importantly, we have shown that EGR2 is critical for Th1 differentiation and that inhibition of EGR2 in vitro suppresses IFNγ production only in MRL-*lpr* lupus CD4^+^ T cells, but not control MRL CD4^+^ T cells. Although further experiments are needed to elucidate the role of EGR2 in the regulation of immune cell development and function in vivo in lupus, our data suggests that EGR2 may function differentially in specific autoimmune lupus context when compared to its role in physiological context.

## Methods

### Human peripheral blood mononuclear cells (PBMCs)

The PBMCs of human patients of lupus (*n* = 4, all female) and healthy controls (*n* = 4, 2 male and 2 female) were purchased directly from AllCells LLC (Alameda, CA, USA). Based on the information provided by the company, three out of four patients had lupus for over 9 years and there is no record of the duration of lupus for the fourth patient. The available donors’ information was summarized and showed in the Supplemental Table 1.

### Mice

Genetically lupus-prone MRL-*lpr* (MRL/MpJ-*Fas*^*lpr*^/J, Stock Number 000485), B6-*lpr* (B6.MRL-*Fas*^*lpr*^/J, Stock Number 000482), B6.*sle123* (B6.NZMSle1/Sle2/Sle3*,* stock #007228), and control MRL *(*MRL/MpJ, Stock Number 000486), B6 (C57BL/6j, stock# 000664) breeders were purchased from The Jackson Laboratory, ME, USA and bred in house. For MRL, MRL-*lpr*, and B6.*sle123* strains, only female mice were used. For B6 and B6-*lpr* stains, both male and female mice were used in this study. All mice were housed in our Association for Assessment and Accreditation of Laboratory Animal Care **(**AAALAC)-certified animal facility at the Virginia-Maryland College of Veterinary Medicine (VMCVM), Virginia Tech. Mice were fed with a commercial 7013 NIH- 31 Modified 6% Mouse/Rat Sterilizable Diet (Harlan Laboratory, Madison, WI, USA) and gave water ad libitum. The mice at designated age were euthanized by CO2 asphyxiation, and spleen tissues were collected for cell preparation and experimental assays. All the experiments were performed in vitro with the cells prepared from the mice. The age and number of animals used for each experiment were given in the corresponding figure legend.

### Splenic cell preparation and total CD4^+^ T cell isolation

Whole splenocytes were prepared according to the standard lab procedures that have been extensively described in detail previously [29–31]. Briefly, the spleen tissue was dissociated by gently scraping through a size 100 mesh steel screen (Sigma-Aldrich, St. Louis, MO, USA), and the cell suspension was passed through a 70-μm cell strainer to remove tissue debris. After lysing red blood cells with ACK-Tris-NH_4_Cl buff, the freshly-isolated splenocytes were pelleted, and adjusted to 5 × 10^6^/ml complete phenol red-free RPMI-1640 medium (HyClone, Inc., Manassas, VA, USA) that was supplemented with 10% charcoal-stripped fetal bovine serum (Atlanta Biologicals, Flowery Branch, GA, USA), 2 mM L-glutamine (HyClone), 100 IU/ml penicillin and 100 μg/ml streptomycin (HyClone), and 1% non-essential amino acids (HyClone) for culture. Aliquots of splenocytes were pelleted and stored at − 80 **°**C for experimental analysis later. Splenic CD4^+^ T cells were purified from freshly-isolated splenocytes using CD4 (L3T4) MicroBeads (Miltenyi Biotec, Auburn, CA, USA) by positive selection per the manufacturer’s instruction. The purity of isolated CD4^+^ T cells (over 90%) was confirmed by flow cytometry after staining the isolated cells with eflour 450-conjugated anti-CD4 antibody (eBioscience/ThermoFisher Scientific, Asheville, NC, USA).

### siRNA transfection

The EGR2 specific Dicer substrate siRNA (EGR2 Dsi) and negative control DsiRNA (NC) were purchased from Integrated DNA Technologies (IDT, Coralville, IA, USA) and reconstituted with distilled water to a stock concentration at 10 μM, then aliquoted and stored at − 80 °C. The DsiRNAs were transfected into splenocytes with Lipofectamine® RNAiMAX Transfection Reagent (ThermoFisher Scientific) per the manufacturer’s instruction. Briefly, Freshly- isolated splenocytes were adjusted to 5 × 10^6^/ml with complete RPMI-1640 medium without penicillin/ streptomycin and seeded in 48 well cell culture plates (250ul cells per well). For each transfection, 0.25 μl DsiRNA from stock solution was mixed 25 μl opti-MEM medium (ThermoFisher Scientific); and 0.5 μl RNAiMAX reagent was diluted in 25 μl opti-MEM medium in a separate tube and incubated at room temperature for 5–10 min. After incubation, the diluted DsiRNA and RNAi/MAX solutions were mixed together (totally will be 50 μl) and applied to the seeded cells (2.5 pmol of DsiRNA per well). Forty eight hours after transfection, the transfected cells were stimulated PMA (Sigma-Aldrich*,* St Louis, MO, USA, 50 ng/ml), ionomycin (Sigma, 1 μg/ml) and BD GolgiPlug protein transporter inhibitor (1x, Fisher Scientific, Suwanee, GA, USA) for 5 h and then collected for intracellular Flow cytometry analysis.

### RT-qPCR

Total RNAs were prepared from splenocytes and CD4^+^ T cells with RNeasy Mini kit. RT-qPCR was performed with iTaq one-step RT-PCR with SYBR green kit (Bio-Rad, Hercules, CA, USA) to quantify the expression of EGR2 as previous described [32, 33]. The EGR2 expression was normalized to housekeeping gene β-actin or 18 s RNA. The data was shown as relative expression level to an appropriate control by using the 2^−ΔΔCt^ formula. QuantiTect 10x qPCR primer mixes for human and mouse β-actin were purchased from Qiagen. The qPCR primers for human, mouse EGR2 and human 18 s were designed with PrimerQuest Tool and synthesized by IDT. The qPCR primer sequences for human EGR2: forward 5′-CTT TGA CCA GAT GAA CGG AGT G-3′ and reverse 5′-AGC AAA GCT GCT GGG ATA TG-3′; mouse EGR2: forward 5′-CTACCCGGTGGAAGACCTC-3′ and reverse 5′-AATGTTGATCATGCCATCTCC-3′; mouse 18 s forward 5′-GCC CTG TAA TTG GAA TGA GTC CAC TT-3′ and reverse 5′-CTC CCA AGA TCC AAC TAC GAG CTT T-3′.

### Intracellular flow cytometry

The eBioscience Foxp3 transcription factor staining buffer set was used for intracellular antigen flow cytometry by following the protocol recommended by the manufacturer. Briefly, the cells were stained with surface marker (CD4, CD8, CD3, B220, CD19 and/or CD44) in MACS buffer (Phosphate-buffered saline (PBS, pH 7.2) supplemented with 0.5% bovine serum albumin (BSA) and 2 mM EDTA), washed, fixed with 1x Foxp3 Fixation/Permeabilization solution for 45 min. The fixed cells were washed with 1x permeabilization buffer and resuspended in 1x permeabilization buffer for intracellular antigen stain with fluorescent conjugated anti-mouse EGR2 (ThermoFisher Scientific) /or anti-mouse IFNγ (ThermoFisher Scientific) antibodies for 45 min. The stained cells were washed and visualized using a FACS Aria flow cytometer (BD Biosciences). The flow data were analyzed with FlowJo version 10 software.

### Th1 polarization

Naïve CD4^+^ T cells were purified from the splenocytes of 6–8-week-old MRL and MRL-*lpr* mice by negative selection using a mouse naïve CD4^+^T cells isolation kit from Miltenyi Biotec. The purity of naïve CD4^+^ T cells was determined by staining the cells with surface maker CD4 and CD44 and characterized as CD4^+^CD44^−^. The naïve CD4 T cells were transfected with EGR2 and NC DsiRNA. Twenty-four hours after DsiRNA transfection, the transfected naïve CD4^+^ T cells were cultured at Th1 polarization condition (plate-bound anti-CD3 (2 μg/ml), anti-CD28(1 μg/ml, IL-2 (5 ng/ml), IL-12 (10 ng/ml) and anti-IL4 (10 μg/ml)) for 3 days. The culture supernatant was collected for IFNγ enzyme-linked immunosorbent assay **(**ELISA). The cells were stimulated with PMA (50 ng/ml), ionomycin (1 μg/ml) and BD GolgiPlug protein transporter inhibitor for 5 h for intracellular flow cytometry analysis.

### Elisa

The level of IFNγ in culture supernatant was determined by standard ELISA procedure. The capture IFNγ and biotin-conjugated detection antibody were purchased from BD Biosciences. The HRP Streptavidin was purchased from Biolegend (San Diego, CA, USA). The KPL SureBlue TMB substrate was purchased from SeraCare Life Science Inc. (Gaithersburg, MD, USA).

### Statistical analysis

All values in the graphs are presented as means ± SEM. Two-tailed, unpaired *t* tests were performed to assess the statistical significance of gene expression between two biological groups (healthy control vs human lupus patient, MRL vs MRL-*lpr*, B6 vs B6-*lpr*, B6 vs B6.*sle123*). Paired *t* tests were performed to assess the statistical significance between NC DsiRNA and specific EGR2 DsiRNA treated samples. *, **, and *** denote *p* < 0.5, *p* < 0.01, and *p* < 0.001 of the statistical significances determined by student *t*-test respectively. One-way ANOVA with Tukey- Kramer all pair’s comparisons were performed to assess the statistical significance among the multiple groups. The means of the biological groups connected with different letter are significantly different. The JMP software (Pro10) from SAS Institute Inc. (Cary, NC, USA) was used for statistical analysis.

## Supplementary information

**Additional file 1: Supplemental Table 1**. Summary of human PBMCs donors’ information. **Supplemental Figure 1.** The EGR2 expression hierarchy in different splenic cell subsets of MRL and MRL-*lpr* mice. The freshly-prepared (t0, resting state) and 24 h of anti-CD3 and anti-CD28 stimulated splenocytes from MRL-*lpr* mice at diseased stage (15 weeks-of age) and age-matched control MRL mice were stained with different cell surface marker (CD4, CD8, CD19, B220, CD3) and then intracellular flow stain of EGR2. (A) The representative histogram plots show the expression of EGR2 in gated DNT, CD4^+^ T, CD8^+^ T, CD19^+^ B cells in resting (t0) and activated (anti-CD3/CD28) MRL splenocytes. (B&C). The summary graphs show EGR2 expression intensity (MFI) in gated specific cell subsets of MRL splenocytes at resting (B) and activated state (C). (D) The representative histogram plots show the expression of EGR2 in gated DNT, CD4^+^ T, CD8^+^ T, CD19^+^ B cells in resting (t0) and activated (anti-CD3/CD28) MRL-*lpr* splenocytes. (E&F) The summary graphs show EGR2 expression intensity in gated specific cell subsets of MRL-*lpr* splenocytes at resting (E) and activated state (F). One-way ANOVA with Tukey- Kramer all pair’s comparisons were performed for statistical analysis of multiple groups comparison. The means of the groups that were not connected with the same letter were significantly different. Two tail, unpaired student *t*-tests were performed for two group comparison (CD4^+^ T vs CD8^+^ T, CD8^+^ T vs CD19^+^ B, DNT vs CD19^+^ B); *, *p* < 0.05. **Supplemental Figure 2.** The EGR2 expression hierarchy in different splenic cell subsets of B6 and B6.*sle123* mice. The freshly-prepared (t0, resting state) and 24 h of anti-CD3 + anti-CD28 stimulated splenocytes from 31 to 32-week-old B6 and B6.*sle123* were stained with different cell surface marker (CD4, CD8, B220), and then intracellular flow stain of EGR2. (A, B) The summary graphs show EGR2 expression intensity in gated specific cell subsets of B6 splenocytes at resting (A) and activated state (B). (C, D) The summary graphs show EGR2 expression intensity in gated specific cell subsets of B6.*sle123* splenocytes at resting (C) and activated state (D). One-way ANOVA with Tukey- Kramer all pair’s comparisons were performed for statistical analysis of multiple groups comparison. The means of the groups that were not connected with the same letter were significantly different. Two tail, unpaired student *t*-tests were performed for two group comparison (CD4^+^ T vs CD8^+^ T, CD8^+^ T vs B220^+^ B, CD4^+^ T vs B220^+^ B); *, *p* < 0.05.

## Data Availability

The datasets used and/or analysed during the current study are available from the corresponding author on reasonable request.

## Abbreviations

DNT: Double negative T cell (CD4^−^CD8^−^CD3^+^B220^+^)
Dsi RNA: Dicer substrate siRNA
EGR2: Early growth response 2
ELISA: Enzyme-linked immunosorbent assay
NC: Negative control
PBMC: Peripheral blood mononuclear cell
MRL-*lpr*: MRL/MpJ-*Fas*^*lpr*^/J
B6-*lpr*: B6.MRL-*Fas*^*lpr*^/J
B6.*sle123*: B6.NZMSle1/Sle2/Sle3
