# Supplementary material for: EGR2 is elevated and positively regulates inflammatory IFNγ production in lupus CD4+ T cells
Source: BMC Immunol. 2020 Jul 9;21:41. doi: 10.1186/s12865-020-00370-z (PMC7346656; doi:10.1186/s12865-020-00370-z)
Supplement: Supplementary file 1 — Additional file 1: Supplemental Table 1. Summary of human PBMCs donors’ information. Supplemental Figure 1. The EGR2 expression hierarchy in different splenic cell subsets of MRL and MRL-lpr mice. The freshly-prepared (t0, resting state) and 24 h of anti-CD3 and anti-CD28 stimulated splenocytes from MRL-lpr mice at diseased stage (15 weeks-of age) and age-matched control MRL mice were stained with different cell surface marker (CD4, CD8, CD19, B220, CD3) and then intracellular flow stain of EGR2. (A) The representative histogram plots show the expression of EGR2 in gated DNT, CD4+ T, CD8+ T, CD19+ B cells in resting (t0) and activated (anti-CD3/CD28) MRL splenocytes. (B&C). The summary graphs show EGR2 expression intensity (MFI) in gated specific cell subsets of MRL splenocytes at resting (B) and activated state (C). (D) The representative histogram plots show the expression of EGR2 in gated DNT, CD4+ T, CD8+ T, CD19+ B cells in resting (t0) and activated (anti-CD3/CD28) MRL-lpr splenocytes. (E&F) The summary graphs show EGR2 expression intensity in gated specific cell subsets of MRL-lpr splenocytes at resting (E) and activated state (F). One-way ANOVA with Tukey- Kramer all pair’s comparisons were performed for statistical analysis of multiple groups comparison. The means of the groups that were not connected with the same letter were significantly different. Two tail, unpaired student t-tests were performed for two group comparison (CD4+ T vs CD8+ T, CD8+ T vs CD19+ B, DNT vs CD19+ B); *, p < 0.05. Supplemental Figure 2. The EGR2 expression hierarchy in different splenic cell subsets of B6 and B6.sle123 mice. The freshly-prepared (t0, resting state) and 24 h of anti-CD3 + anti-CD28 stimulated splenocytes from 31 to 32-week-old B6 and B6.sle123 were stained with different cell surface marker (CD4, CD8, B220), and then intracellular flow stain of EGR2. (A, B) The summary graphs show EGR2 expression intensity in gated specific cell subsets of B6 splenocytes at restin [file 12865_2020_370_MOESM1_ESM.docx]

| **Sample ID** | **Gender** | **Age (years)** | **duration of disease** | **Race** | **Medicine** | **disease activity score (DAS28)** |
| --- | --- | --- | --- | --- | --- | --- |
| Healthy control #1 | Female | 31 | N/A | Non-Hispanic White | VD3, Biotin | N/A |
| Healthy control #2 | Male | 40 | N/A | Non-Hispanic White | No | N/A |
| Healthy control #3 | Male | 28 | N/A | Non-Hispanic White | No | N/A |
| Healthy control #4 | Female | 31 | N/A | Asian | NO | N/A |
| Lupus patient #1 | Female | 51 | 9 years | Non-Hispanic White | multiple medicine | 6 |
| Lupus patient #2 | Female | 51 | 9 years | Hispanic | Plaquenil/Azathioprine | 6 |
| Lupus patient #3 | Female | 46 | 14 years | Non-Hispanic White | Plaquenil/ prednisone | N/A* |
| Lupus patient #4 | Female | 24 | N/A | N/A | N/A | N/A |

* This patient has a RF test result at 99iu/ml.

**Supplemental Table 1:** Summary of human PBMCs donors’ information.


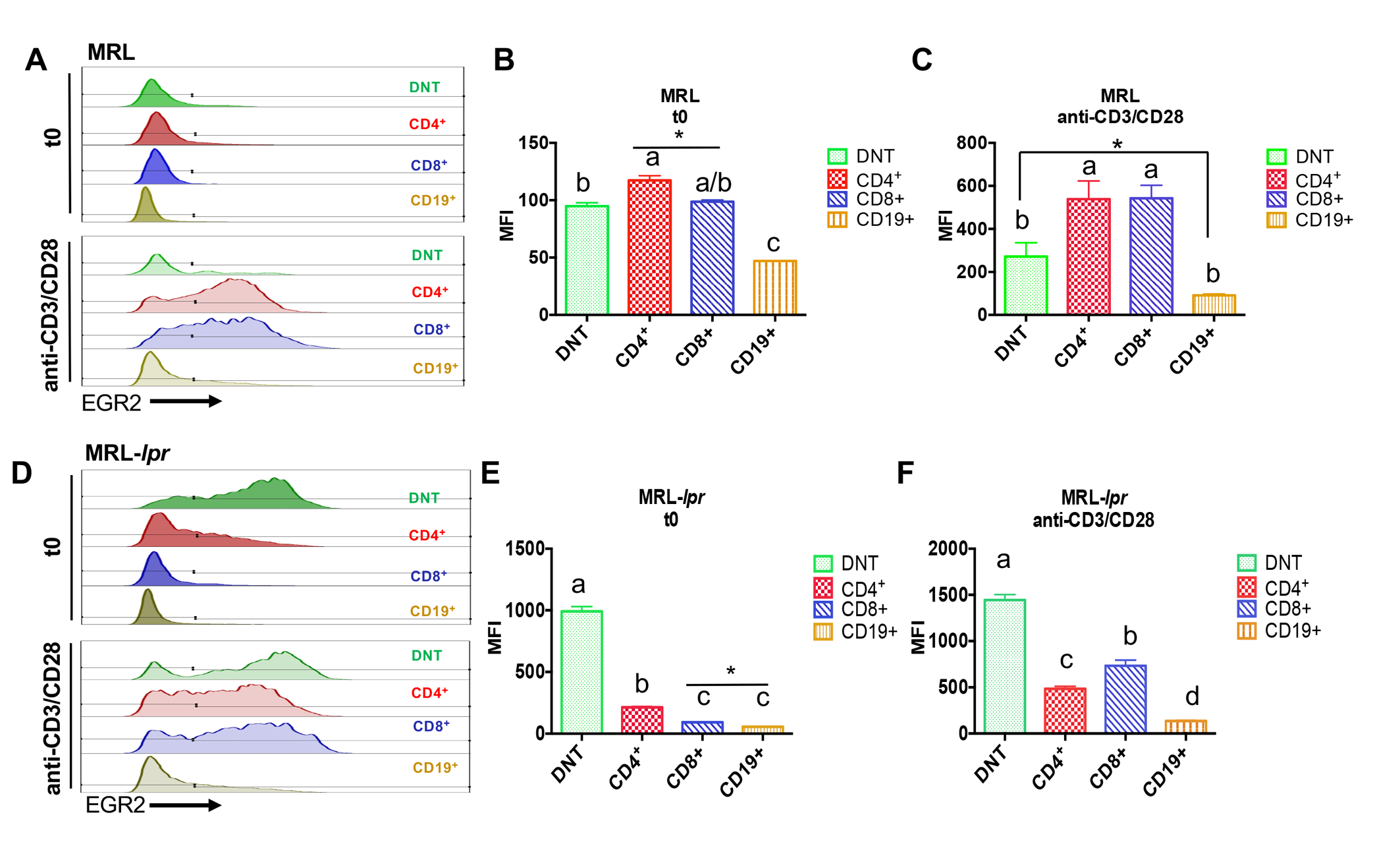


**Supplemental Figure 1: The EGR2 expression hierarchy in different splenic cell subsets of MRL and MRL-*lpr* mice.** The freshly-prepared (t0, resting state) and 24 hours of anti-CD3 and anti-CD28 stimulated splenocytes from MRL-*lpr* mice at diseased stage (15 weeks-of age) and age-matched control MRL mice were stained with different cell surface marker (CD4, CD8, CD19, B220, CD3) and then intracellular flow stain of EGR2. (**A**) The representative histogram plots show the expression of EGR2 in gated DNT, CD4^+^ T, CD8^+^ T, CD19^+^ B cells in resting (t0) and activated (anti-CD3/CD28) MRL splenocytes. (**B&C**). The summary graphs show EGR2 expression intensity (MFI) in gated specific cell subsets of MRL splenocytes at resting (B) and activated state (C). (**D**) The representative histogram plots show the expression of EGR2 in gated DNT, CD4^+^ T, CD8^+^ T, CD19^+^ B cells in resting (t0) and activated (anti-CD3/CD28) MRL-*lpr* splenocytes. (**E&F**) The summary graphs show EGR2 expression intensity in gated specific cell subsets of MRL-*lpr* splenocytes at resting (E) and activated state (F). One-way ANOVA with Tukey- Kramer all pair's comparisons were performed for statistical analysis of multiple groups comparison. The means of the groups that were not connected with the same letter were significantly different. Two tail, unpaired student *t*-tests were performed for two group comparison (CD4^+^ T *vs* CD8^+^ T, CD8^+^ T *vs* CD19^+^ B, DNT *vs* CD19^+^ B); *, *p*<0.05.


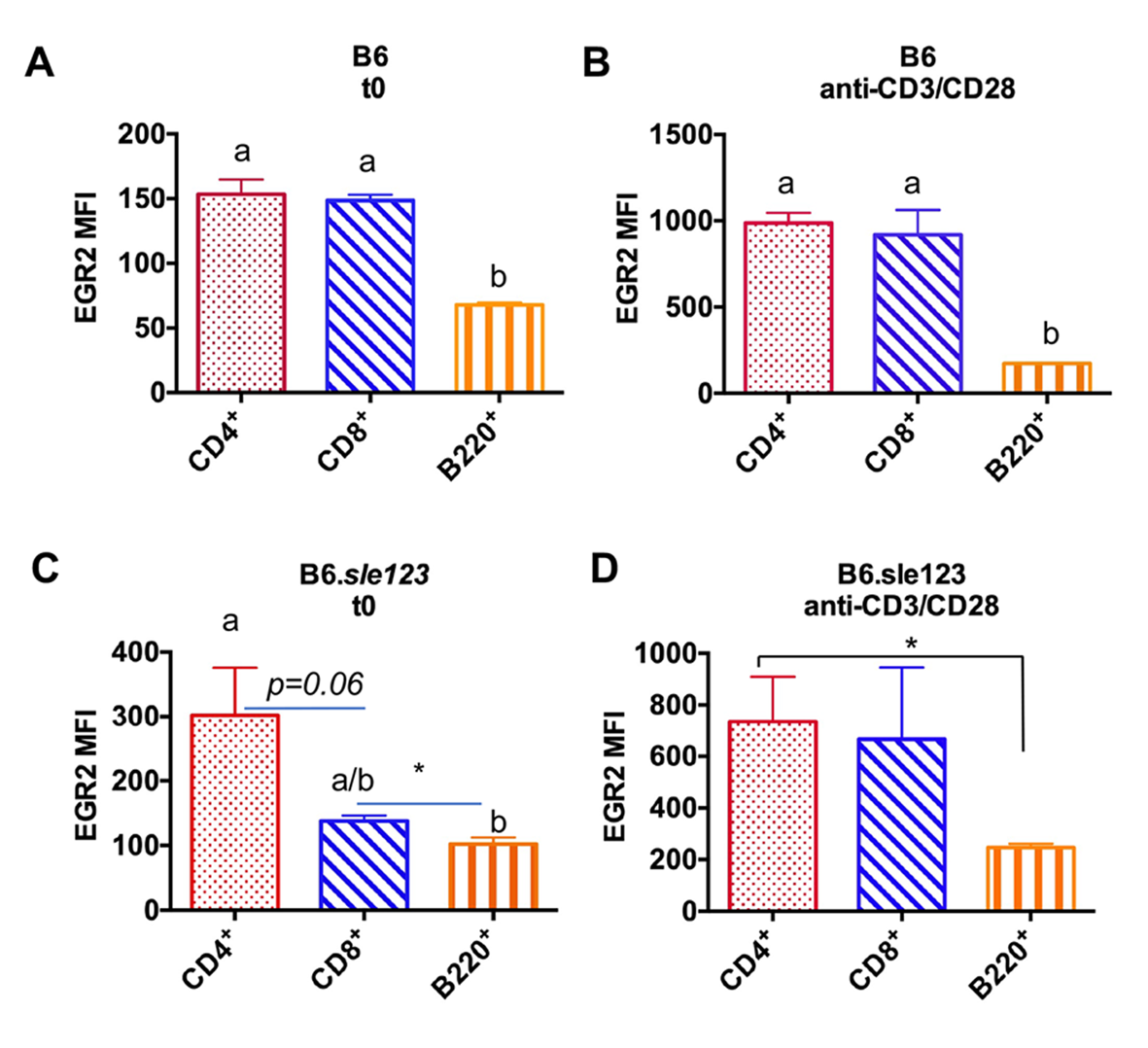
**Supplemental Figure 2: The EGR2 expression hierarchy in different splenic cell subsets of B6 and B6.*sle123* mice.** The freshly-prepared (t0, resting state) and 24 hours of anti-CD3+anti-CD28 stimulated splenocytes from 31-32-week-old B6 and B6.*sle123* were stained with different cell surface marker (CD4, CD8, B220), and then intracellular flow stain of EGR2. **(A, B**) The summary graphs show EGR2 expression intensity in gated specific cell subsets of B6 splenocytes at resting (A) and activated state (B). (**C, D**) The summary graphs show EGR2 expression intensity in gated specific cell subsets of B6.*sle123* splenocytes at resting (C) and activated state (D). One-way ANOVA with Tukey- Kramer all pair's comparisons were performed for statistical analysis of multiple groups comparison. The means of the groups that were not connected with the same letter were significantly different. Two tail, unpaired student *t*-tests were performed for two group comparison (CD4^+^ T *vs* CD8^+^ T, CD8^+^ T *vs* B220^+^ B, CD4^+^ T *vs* B220^+^ B); *, *p*<0.05.
